# Editorial: Recent advances in obstructive sleep apnoea (OSA)

**DOI:** 10.3389/fmed.2025.1609695

**Published:** 2025-05-09

**Authors:** Ling Zhou, Lingling Wang, Pengdou Zheng, Xiaoyan Zhu, Guisha Zi, Lixiang Chen, Xiaojing Cai, Huiguo Liu, Wei Liu

**Affiliations:** ^1^Department of Respiratory and Critical Care Medicine, Key Laboratory of Pulmonary Diseases of Health Ministry, Tongji Hospital, Tongji Medical College, Huazhong University of Science and Technology, Wuhan, Hubei, China; ^2^Department of Geriatrics, Tongji Hospital, Tongji Medical College, Huazhong University of Science and Technology, Wuhan, Hubei, China; ^3^Key Laboratory of Vascular Aging, Ministry of Education, Tongji Hospital, Tongji Medical College, Huazhong University of Science and Technology, Wuhan, Hubei, China

**Keywords:** obstructive sleep apnea, bioinformatics, multidisciplinary approach, translational research, cardiovascular-metabolic comorbidity

## Introduction

Obstructive sleep apnea (OSA), a chronic respiratory disorder characterized by recurrent upper airway collapse during sleep, has emerged as a global health challenge, affecting nearly one billion individuals worldwide. Its prevalence exceeds 50% in certain high-risk populations, underscoring its status as a silent epidemic (1). Beyond its direct respiratory consequences, OSA is intricately linked to a 2–3-fold increased risk of cardiovascular and metabolic diseases, including hypertension, coronary heart disease, diabetes, and cognitive impairment (2). Despite its profound public health burden, therapeutic options remain limited, with continuous positive airway pressure (CPAP) therapy recognized as the gold standard; however, adherence rates are suboptimal (3). The absence of definitive pharmacological interventions and reliable biomarkers further complicates clinical management. This Research Topic, “*Recent Advances in Obstructive Sleep Apnea (OSA)*,” was conceived to address these gaps by fostering interdisciplinary dialogue, highlighting novel methodologies, and translating mechanistic insights into actionable clinical strategies. The 10 articles featured in this Research Topic reflect the diversity and depth of contemporary OSA research. They encompass pathophysiology, genetic and epidemiological associations, biomarker discovery, and therapeutic innovations, collectively advancing our understanding of OSA as a systemic disorder with far-reaching implications. Below, we synthesize these contributions within three thematic frameworks: (1) OSA as a multisystem disease, (2) bioinformatics and genetic insights, and (3) therapeutic advancements and challenges.

OSA as a Multisystem Disease: Unraveling Complex Associations recurring theme across this Research Topic is the recognition of obstructive sleep apnea (OSA) as a systemic condition intertwined with a variety of comorbidities. Liu P. et al. explore the bidirectional relationship between OSA and polycystic ovary syndrome (PCOS), positing shared hormonal and inflammatory pathways. Their work highlights insulin resistance and hyperandrogenism as potential mediators, suggesting that OSA screening should be prioritized in cohorts with PCOS. Similarly, Zhao et al. employ Mendelian randomization (MR) to disentangle the causal links between hypothyroidism and OSA. Their findings reveal a bidirectional association, implicating thyroid hormone dysregulation in upper airway collapsibility and intermittent hypoxia (IH)-induced thyroid dysfunction. These studies underscore the necessity for holistic management of OSA within the context of endocrine disorders. The systemic impact of OSA extends to musculoskeletal and oncological domains. Research synthesizes evidence linking OSA to osteoarthritis (OA), proposing chronic inflammation and oxidative stress as shared mechanisms (Weng et al.). Meanwhile, Yao et al. combine cohort studies and MR to demonstrate a robust association between OSA and lung cancer risk. Their analysis suggests that IH-driven hypoxia-inducible factor (HIF) activation and immune dysregulation may fuel carcinogenesis, a hypothesis warranting longitudinal validation. Cardiovascular complications, a hallmark of OSA, are revisited by Chen et al. Research who elucidate the role of cardiac autonomic dysfunction and structural remodeling in OSA-related arrhythmias (Chen et al.). Their work emphasizes the need for early autonomic profiling to stratify arrhythmia risk in patients with OSA. Collectively, these articles reinforce OSA's role as a multisystem disruptor, urging clinicians to adopt a proactive, comorbidity-aware approach.

Bioinformatics and Genetic Insights: Decoding OSA's Complexity The integration of bioinformatics and genetic methodologies has revolutionized research on obstructive sleep apnea (OSA), enabling high-throughput discovery of biomarkers and mechanistic pathways. Xie et al. introduce the Intermittent Hypoxia Index, a novel metric for quantifying the severity of intermittent hypoxia. By correlating IHI with markers of endothelial dysfunction and oxidative stress, they provide a framework for personalized risk assessment. This aligns with the findings of Liu, Yang et al., who review pharmacological targets derived from transcriptomic and proteomic analyses, including hypoxia-inducible factor (HIF) inhibitors and anti-inflammatory agents. Genetic epidemiology takes center stage in the work of Zhao et al. and Yao et al., Both leveraging Mendelian randomization (MR) to infer causality-a method that minimizes confounding biases inherent to observational studies. These contributions exemplify how genetic tools can disentangle the etiological web of OSA, identifying modifiable risk factors and therapeutic targets. Research explores the regulatory role of estrogen in the pathogenesis of OSA, integrating gene expression data from animal models and clinical cohorts (Zhou et al.). The authors propose estrogen replacement therapy as a potential intervention for postmenopausal women with OSA, bridging bench-to-bedside innovation. Such findings underscore the transformative potential of bioinformatics in guiding precision medicine.

Therapeutic Advancements and Challenges: Toward Personalized Management Despite the efficacy of Continuous Positive Airway Pressure (CPAP) therapy, its limitations necessitate the exploration of alternative strategies. Seifen et al. investigate periodic limb movements (PLMs) in obstructive sleep apnea (OSA) patients without comorbidities, revealing a subset of patients who experience PLM-driven sleep fragmentation that is resistant to CPAP treatment. Their work advocates for the use of polysomnographic phenotyping to tailor therapeutic interventions. The critical appraisal of pharmacological innovation is presented by Liu, Xu et al., who catalog emerging drug candidates that target pathways induced by intermittent hypoxia (IH). Promising agents identified include leptin analogs and antioxidants; however, clinical trial data remain sparse. Their findings advocate for multidisciplinary care models that integrate expertise from respiratory, endocrine, and nutritional disciplines.

This Research Topic underscores the complexity of OSA while charting a path toward translational solutions. Key takeaways include: 1. OSA serves as a gateway to systemic disease, necessitating focused screening and management of comorbidities. 2. Bioinformatics and genetic tools are indispensable for unraveling the heterogeneity of OSA and identifying relevant biomarkers. 3. Multidisciplinary therapies—combining CPAP, pharmacological agents, and lifestyle interventions—hold promise for mitigating metabolic and cardiovascular sequelae. However, challenges persist. The lack of large-scale omics datasets from diverse populations limits the generalizability of biomarkers. Furthermore, the bidirectional causality between OSA and its comorbidities necessitates longitudinal studies to clarify temporal relationships.

## Conclusion

The articles in this Research Topic exemplify the vibrancy of Obstructive Sleep Apnea (OSA) research, effectively bridging mechanistic discovery and clinical innovation. As guest editors, we express our sincere gratitude to the authors, reviewers, and readers for their invaluable contributions to this evolving field. By fostering collaboration across genetics, bioinformatics, and clinical medicine, we are progressing toward transforming OSA from a widespread burden into a manageable condition.

